# Transmission dynamics of human herpesvirus 6A, 6B and 7 from whole genome sequences of families

**DOI:** 10.1186/s12985-022-01941-9

**Published:** 2022-12-24

**Authors:** Brianna S. Chrisman, Chloe He, Jae-Yoon Jung, Nate Stockham, Kelley Paskov, Dennis P. Wall

**Affiliations:** 1 Department of Bioengineering, Stanford University, Serra Mall, Stanford, USA; 2 Department of Biomedical Data Science, Stanford University, Serra Mall, Stanford, USA; 3 Department of Pediatrics (Systems Medicine), Stanford University, Serra Mall, Stanford, USA; 4 Department of Neuroscience, Stanford University, Serra Mall, Stanford, USA; 5 Nevada Bioinformatics Center, University of Nevada, Reno, USA

**Keywords:** Whole genome sequencing, Blood virome, Human herpesvirus

## Abstract

While hundreds of thousands of human whole genome sequences (WGS) have been collected in the effort to better understand genetic determinants of disease, these whole genome sequences have less frequently been used to study another major determinant of human health: the human virome. Using the unmapped reads from WGS of over 1000 families, we present insights into the human blood DNA virome, focusing particularly on human herpesvirus (HHV) 6A, 6B, and 7. In addition to extensively cataloguing the viruses detected in WGS of human whole blood and lymphoblastoid cell lines, we use the family structure of our dataset to show that household drives transmission of several viruses, and identify the Mendelian inheritance patterns characteristic of inherited chromsomally integrated human herpesvirus 6 (iciHHV-6). Consistent with prior studies, we find that 0.6% of our dataset’s population has iciHHV, and we locate candidate integration sequences for these cases. We document genetic diversity within exogenous and integrated HHV species and within integration sites of HHV-6. Finally, in the first observation of its kind, we present evidence that suggests widespread de novo HHV-6B integration and HHV-7 integration and reactivation in lymphoblastoid cell lines. These findings show that the unmapped read space of WGS is a promising source of data for virology research.

**Supplementary Information:**

The online version contains supplementary material available at 10.1186/s12985-022-01941-9.

## Background

As the cost and speed of whole genome sequencing (WGS) continues to improve, many research institutions have undertaken large scale whole genome sequencing studies in an effort to better understand genetic determinants of human diseases [1–4]. While high coverage (>30x) WGS produces several hundred gigabytes of raw data per sample [5], in many pipelines up to 30% of these reads go unused because they fail to align to the human reference genome. [6]. These unmapped reads may originate from non-reference human DNA sequences, organic reagents and contamination, and human viruses.

Meanwhile, the last decade of advances in sequencing has also empowered the field of metagenomics and the study of the human microbiome, areas where next-gen sequencing technologies have allowed for the rapid characterization of bacteria, small eukaryotes, and viruses that inhabit human environments. While much of microbiome and virome research has focused on the gut microbiome [7, 8] which has clear communication links between the human digestive system, nervous system and immune system, recently it has been suggested that microbiota with low microbial loads may also play novel roles in disease [9–11]. One such microbiota is human blood, and it is still up for debate whether or not there is a healthy blood microbiota or whether the presence of bacteria inherently indicates disease [12–14]. Several studies have investigated the blood bacteriome in an attempt to understand the healthy blood bacterial microbiome, but only a handful studies have attempted to characterize the human blood virome and have been done in mostly diseased cohorts [15–17]. Furthermore, despite the importance of the blood virome in blood transfusion and stem cell transplant safety [18, 19], research in emerging pathogens [20, 21], and immune system regulation [22], to our knowledge only one study has analyzed the blood virome on the scale of thousands of individuals [23].

The early stages of the SARS-CoV-2 pandemic underscored how important it is to understand transmission patterns for different viruses. [24]. Particularly in the case of intrafamilial transmission, non-sexual transmission between members of the same household, a better understanding of such might help families take steps to mitigate the risk of infection. Several known bloodborne viruses with high disease risk, such as betapapillomaviruses and hepatitis C [25–27], show evidence of intrafamilial transmission. Furthermore, some human herpesviruses (HHV) have the ability to integrate into host genomes, and ancient integration events of human herpesvirus 6A and human herpesvirus 6B have persisted as a relatively common genotype, displaying Mendelian inheritance patterns. The prevalence and integration patterns of herpes 6A and 6B are not yet fully understood, though inherited chromosomally integrated herpesviruses (iciHHV) may place a role in cardiovascular disease [28, 29].

The iHART dataset [30] contains whole genome sequences from whole blood (WB) or lymphoblastoid cell lines (LCLs) from over 4500 individuals from over 1000 different nuclear families with multiplex autism. Originally curated to understand the genetic determinants of autism, the iHART dataset has become valuable not only for autism research, but because its unique family structure allows for understanding of inheritance patterns that cannot be done with case-control cohorts [31–34]. In this study, we utilize family structure to better understand intra-family viral transmission and integration patterns. We characterize the human blood DNA virome, focusing particularly on intra-family transmission patterns and chromosomal integration and inheritance of herpesviruses.

## Results

### Unmapped read space characterizes prevalence and abundance of viruses

**Fig. 1:**
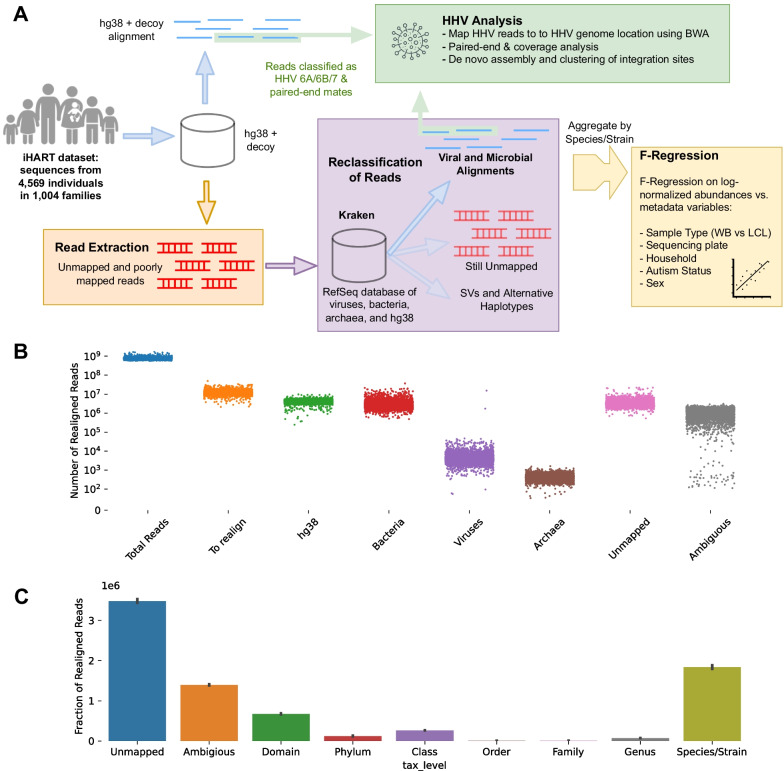
**A** General workflow of the study. Poorly mapped and unmapped reads were extracted from the iHART dataset, reclassified to a database of bacteria, archaea, and GRCh38, and aggregated over species. Reads that were classified as human herpesvirus 6A, 6B, or 7 were mapped to the HHV genomes and analyzed in conjunction with their mates. **B** Number of total and poorly mapped or unmapped reads per sample, and the distributions of their reclassification phyla. **C** Phyla of Kraken reclassifications. Because Kraken classified the majority of reads down to the species level, we aggregated read counts by species/strain

Using unmapped or poorly aligned reads from WGS of 4568 individuals (Fig. 1), we were able to reclassify reads to over 100 species of viruses. We show the top 50 most abundant viruses in Additional file 1: Fig. S1, clustered by Spearman association across samples. Of note, we see four important categories of viruses: Human herpesviruses (HHV) 6A, 6B, and 7 are common blood viruses that are normally acquired during childhood. HHV-6 has the ability to integrate into host cells, and can be inherited through ancient integration events in germline cells that are passed down mendellianly. HHV-6A, 6B, and 7 viral reads are likely true HHVs present in the blood, and we discuss the viral load profiles of HHV-6 and HHV-7 in depth later on. Lambda phage PhiX is a common reagent used in sequencing pipelines to calibrate Illumina machines and balance GC content. Reads classified as PhiX relatives are probably either mismappings to homologous regions, or contamination of the commercial PhiX reagents [23]. Similarly, Epstein Barr virus (EBV) or human gammaherpesvirus 4, is used to immortalize lymphoblastoid cell lines (LCLs). While small amounts of endogenous EBV, the virus that causes mononucleosis, can be present in human blood, our samples has levels of EBV too high to be consistent with endogenous EBV and in abundances consistently higher in the LCL samples (Additional file 1: Fig. S1B). Therefore, EBV and relatives are probably artifacts from the LCL immortalization pipeline. On the other hand, Torque Teno Viruses (TTV) and Erythroviruses are fairly common blood viruses that are usually acquired during childhood, and do not have a strong association with any experimental variables, as we will discuss shortly. We therefore suspect the TTV and erythrovirus reads are probably true reads originating from an active TTV or erythrovirus infection.

Using an F-regression, and regressing viral load against sequencing plate, biological sample source (WB vs LCL), and sample metadata (such as autism phenotype, sex, household/family, and parent vs. child status), we identified several viruses significantly associated with sequencing plate (Additional file 1: Fig. S1B), biological sample type (Additional file 1: Fig. S1D–E), as previously described in [34], and household/family (Additional file 1: Fig. S1C).

Interestingly, we found several viruses associated with household/family, indicating that family members may be transmitting an active infection within their household. Even in low counts, we see a statistically significant family association for torque teno virus (adj. *p* value in F-regression < .05), as well as for erythroparvovirus. This suggest that TTV and erythroviruses, which are commonly acquired during childhood, may frequently be transmitted within a household. We also saw an significant association between family and human herpesviruses. This particular association is likely driven by two mechanisms: primarily, inherited chromosomally integrated human herpesvirus (iciHHV), are passed down from parent to child through Mendelian inheritance, and secondarily, family members transmit active infections.

The family structure of the iHART dataset lends itself well to understanding these inheritance and integration patterns of inherited and acquired human herpesviruses. For the rest of our results and analysis, we focus on the integration and inheritance patterns of human herpesvirus 6A, 6B, and 7. We compute the prevalence of inherited chromsomally integrated human herpesvirus 6 (iciHHV-6), characterize the genetic diversity of inherited and acquired HHV-6 and HHV-7, and identify candidate integration sites of HHV-6. Additionally, we observe a novel integration pattern of HHV-6B and HHV-7 in LCLs - suggesting that HHV-6B can integrate into LCLs and HHV-7 can integrate and reactivate - and hypothesize that this is due to the LCL immortalization process.

### 0.6% of population shows evidence of iciHHV-6

**Fig. 2:**
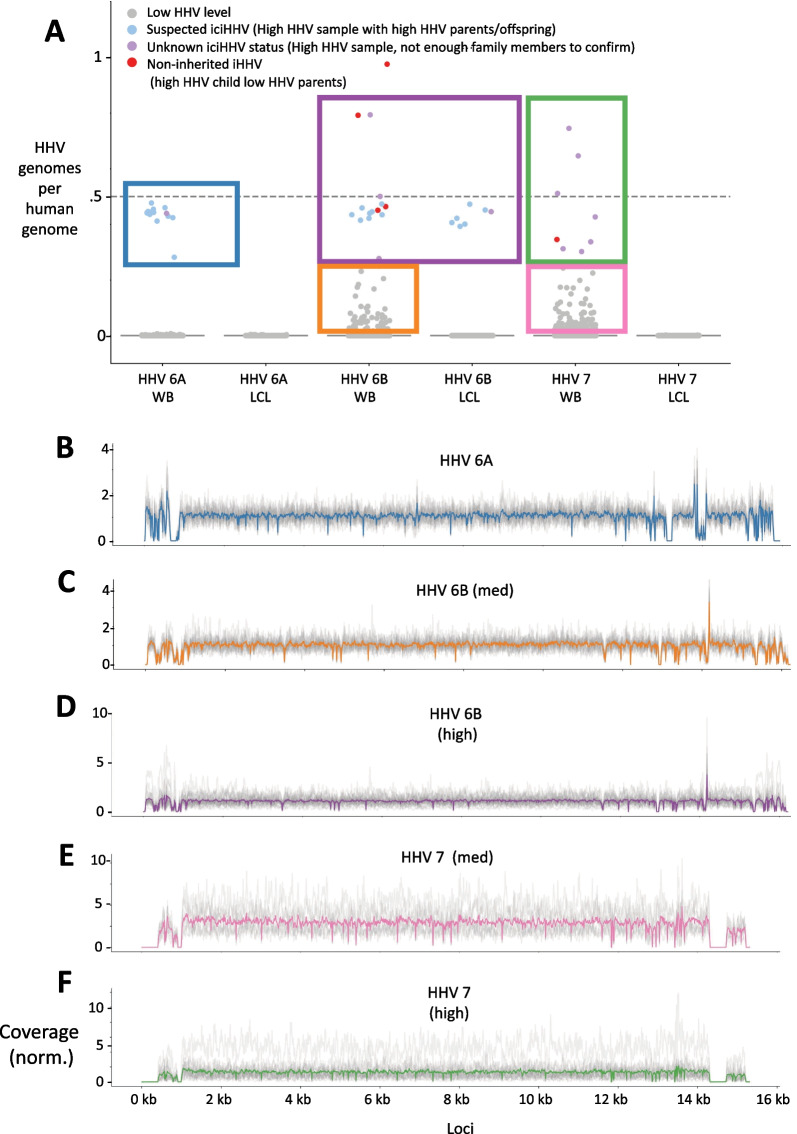
**A** Counts of HHV genomes, normalized by coverage of housekeeping genes ERB and HBB. A normalized HHV genome/human genome fraction of around .5 is consistent with 1 HHV genome per host cell, and an HHV genome/human genome fraction of 1 is consistent with 2 HHV genomes/host cell. Dots represent samples and are colored by observed inheritance patterns, and several groups of interest are boxed. **B**–**H** Normalized coverages of HHV genomes. A normalized coverage of 1 indicates that the expected number of HHV reads under a uniform coverage distribution fall under that region of the genome. Grey lines indicate coverages distributions of each sample, and bold colored lines represent the average. **B** Normalized coverage of HHV-6B from samples with “high 6A” defined by > .25 HHV-6A genomes/human genome. **C** Normalized coverage of HHV-6B from samples with “medium 6B” defined by between .01 and .25 HHV-6B genomes/human genome. **D** Normalized coverage of HHV-6B from samples with “high 6B” defined by > .25 HHV-6B genomes/human genome. **E** Normalized coverage of HHV-7 from samples with “medium 7” defined by between .01 and .25 HHV-7 genomes/human genome. **F** Normalized coverage of HHV-7 from samples with “high 7” defined by > .25 HHV-7 genomes/human genome

Human herpesvirus 6 can integrate into host genomes, and ancient germline integration events can be seen in present day as mendellianly inherited genotypes. We identified 28 samples (.6% of samples, 14 with iciHHV-6A and 14 with iciHHV-6B) that we identified as having a likelihood of iciHHV-6A or iciHHV-6B. These samples had HHV read counts consistent with 1 copy of HHV per cell (or .5 HHV genomes/human genome copy), and had a parent or child in the same family also with high HHV-6A or 6B counts. The HHV counts of these samples and others are shown in Fig. 2A. The probable iciHHV-6B samples came from both WB and LCLs. While all 14 cases of iciHHV-6A were only found LCL samples, the LCL samples outnumber WB by 10-fold so this was not statistically significant. There was no overlap between the samples with likely iciHHV-6A and those with likely iciHHV-6B. Additionally, no samples showed evidence of homozygous iciHHV (a copy of iciHHV inherited from each parent, and therefore 2 copies of iciHHV/human genome).

**Fig. 3:**
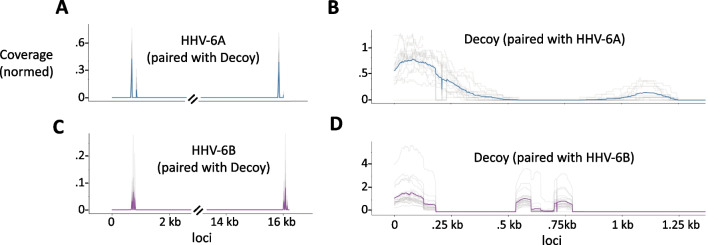
**A**–**D** Paired coverage analysis for HHV-6A and HHV-6B. Grey lines indicate the normalized coverages of each sample, and the bold colored line indicates the average. **A** and **B** were created using samples with at least .25 HHV-6A genomes/host housekeeping genes, and **C** and **D** were created using samples with at least .25 HHV-6B genomes/host housekeeping genes. **A** Normalized coverage of HHV-6A reads that had a mate paired to the decoy reference sequence chrUn_JTFH01000690v1_decoy. A normalized coverage of 1 would indicate that 100% of expected number of reads from a given region of the genome had a mate mapped to the decoy genome. **B** Normalized coverage of HHV-6B reads that had a mate paired to the decoy reference sequence chrUn_JTFH01000690v1_decoy. **C** Normalized coverage of reads mapped to the decoy reference chrUn_JTFH01000690v1_decoy with a mate mapped to HHV-6A sequence. **D** Normalized coverage of reads mapped to the decoy reference chrUn_JTFH01000690v1_decoy with a mate mapped to HHV-6B sequence

Additional evidence for iciHHV-6 came from the coverage profiles of samples with high HHV-6A and high HHV-6B (Fig. 2B, C). We defined “high HHV” to mean at least .25 copies of HHV genome per human genome, suggesting the sample has integrated HHV, or an active infection. Although there are some regions with slightly lower or higher average coverages (corresponding to homologous regions between 6A and 6B, low complexity regions, or high GC content regions), no single gene or region dominated the dominates the coverage profile, indicating that full HHV-6 viral genomes exist in these samples and are not artifacts of mismappings, nor homologous regions between the human genome and HHV. We found similar coverage profiles for HHV-6B with medium viral loads (.01–.25 copies/human genome) (Fig. 2D) and HHV-7 in samples with both high and medium viral loads (Fig. 2E, F).

### Circulating HHV-6B de novo integrates into LCLs

HHV-6A abundance showed a clear bimodal distribution, with a handful of samples with abundances consistent with iciHHV (“high 6A” samples in Fig. 2A), and the rest with very low abundances of HHV-6A (consistent with no viral load, or a latent infection). In the whole blood samples, HHV-6B behaves similarly. However, HHV-6B showed a different pattern in the LCLs. In the LCLs, HHV-6B had a more continuous distribution in the LCLs, with many samples having abundances >0.01 copies/genome reads but not showing inheritance patterns consistent with iciHHV (“med 6B” samples in Fig. 2A). We hypothesized that in these samples, HHV-6B has de novo integrated, a process that has been observed in a laboratory cell culture settings for HHV-6 [35–39]. Thus, we note that these medium abundance HHV-6B reads do seem to be coming from the HHV-6B genome, and not from artifacts or mismappings: coverage profiles suggest only minimal mismappings between HHV-6A and HHV-6B at conserved regions, and no mismappings from or correlation to gammaherpesvirus 4 (Additional file 1: Fig. S2).

To further investigate our hypothesis of de novo HHV 6B integration, we used the paired-end nature of our reads to look for mates between HHV ends, and the human genome, that may point to a specific integration site within the human genome. As expected for integrated viruses, we found that reads mapped to the end of HHV-6A and HHV-6B frequently had a mate mapped to the human genome. Specifically, the mates of both the HHV-6A and 6B ends often mapped to a region in the decoy reference genome, chrUn_JTFH01000690v1_decoy (Fig. 3A–D). This reference sequence is probably an unplaced telomeric sequence, and serves as a HHV integration site. Using these reads, we de novo assembled and clustered the potential integration sites in order to further validate our de novo integration hypothesis, by characterizing specific human genome sequences where de novo integration of HHV-6B might occur. We saw a number common integration sequences for HHV-6A and HHV-6B. In HHV-6B, both probable iciHHV-6A and 6B and de novo integrated HHV-6B samples share 3 canonical integration sequences. We discuss the genetic diversity of these integration sequences later on, when we identify candidate integration sites for HHV-6.

These possible de novo integration events occurred only in LCLs, and therefore we hypothesize that the LCL immortalization process primes the telomeric ends of the human chromosomes to allow for de novo integration of HHV. LCLs are immortalized using the related gammaherpesvirus 4 (the Epstein–Barr virus), which may destabilize the telomeric ends of chromosomes when establishing latency and immortalizing the cell. HHV-6 does not play any intentional role in the LCL pipeline, nor did we find any relationship between EBV and HHV-6B viral loads that might indicate contamination between EBV and HHV-6B or mismappings. Rather, we hypothesize that by telomerase activity and homeostasis at the telomeres [40, 41], EBV allows for integration of HHV-6B into the telomeres. Therefore, a plausible explanation for this spectrum of HHV-6B abundance is that HHV-6B is chromosomally integrated with a fraction of the cells in the sample: During a HHV-6B infection, HHV-6B established latency via integration one or more lymphocytes. Genetic drift, natural selection, or reactivation during that person’s life and during LCL passaging causes different samples to have different fractions of infected cells. It is unclear if HHV-6A cannot achieve this same de novo integration, or if acquired HHV-6A is simply so much less common [42] that we do not find enough cases of acquired HHV-6A in our dataset to verify its de novo integration capabilities.

### Circulating HHV-7 de novo integrates and reactivates in LCLs

In agreement with the literature, which has yet to find a case of inherited HHV-7, we did not find any evidence of iciHHV-7 in our data: no WB cells had high counts of HHV-7, and none of the LCL samples with HHV-7 counts consistent with iciHHV had parent-offspring relationships. This is consistent with findings that HHV-6, but not HHV-7, can commonly integrate into chromosomes of host germline cells [43]. However, like HHV-6B, HHV-7 shows a continuous distribution in the LCLs (Fig. 2A “high 7” and “med 7” samples), suggesting that the immortalization process also affects HHV-7 integration, latency, and reactivation. We hypothesize that like HHV-6B, HHV-7 also de novo establishes latency by integrating into the chromosomes of LCLs during immortalization. Then, unlike HHV-6B, HHV-7 reactivates and maintains itself in extrachromsomal form outside of the human chromosomes.

Many studies have established that HHV-6 establishes latency via integrating in to the human telomeres. HHV-7 shares many similar genomic regions to HHV-6A and HHV-6B, including the direct repeats that facilitate integration. Although HHV-7 integration has not been observed at scale before, a recent study found a likely case of integrated HHV-7, and also determined that laboratory primers may not accurately detect clinical strains of HHV-7, limiting previous experiments’ ability to detect iciHHV-7 [44]. We therefore hypothesize that these medium levels of HHV-7 resulted from an integration event in the LCLs.

However, on the other hand, unlike HHV-6A and HHV-6B, HHV-7 reads did not consistently pair with any GRCh38 or decoy contigs, nor did they frequently pair with unmapped reads. Notably, the coverage profiles of HHV-7 were missing regions within the direct repeats (DRs) of HHV-7 (the 10,000 bps of each end of the 153,000 bp-long assembly) (Fig. 2D, E). Recent studies have shown that when human herpesviruses (HHV-6A in the study) integrate and then reactivate, they lose their DRs in the process. Therefore, this pattern in our data suggests that HHV-7 viruses integrated into the genome of LCL samples at some point and then reactivated, and are now in episomal form [45].

We ran the same HHV alignment pipeline on unmapped reads from the 1000genomes dataset [46] of high coverage WGS from around the world. Interestingly, we did not find a continuous distribution of HHV-6B; rather we found a bimodal distribution with most samples having almost 0 HHV-6B read counts, and <1% of samples having HHV-6B read counts consistent with HHV-6B. In the 1000genomes cohort, also WGS derived from LCLs, we also found only one case of medium abundance HHV-6B (500 reads), with the rest of the samples having <10 reads aligning to HHV-7. Notably, we found HHV-7 and HHV-6B to be more abundant in children than parents in our dataset. Because the 1000genomes data we used was all from adults, we hypothesize that childhood infection (coupled with de novo integration of HHV-6B and HHV-7 into LCLs) is driving the odd distributions of HHV-6B and HHV-7 in the iHART dataset. Alternatively, the immortalization and storage processes in the iHART dataset may be increasing integration and intra-sample re-infection rates.

### HHV displays genetic diversity across hosts

**Fig. 4:**
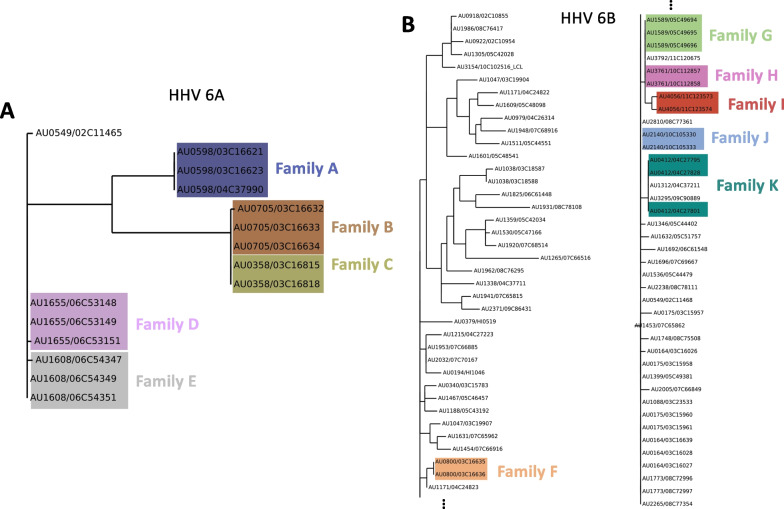
Phylogenetic tree of de novo assembled HHV 6A (**A**) and 6B (**B**) genomes from the samples where de novo assembly was successful. Leaves are labelled with family and sample IDs <FAMILY ID>/<SAMPLE ID>. Suspected cases of iciHHV are highlighted, with coloring according to <FAMILY ID>

We wished to understand the origins and diversity of circulating, latent, and iciHHV. We de novo reconstructed the HHV genome from each sample when possible (de novo assembly failed for samples with low HHV read counts) , and compared genomes using MAAFT multiple sequence alignment and ClustalW phylogenetic tree generation (See Methods). As seen in Fig. 4 and Additional file 1: Fig. S2, HHV 6A, 6B and 7 exhibit genetic diversity across our samples. HHV 6A genomes fall into three distinct clusters (Fig. 4A). Family members always fall into the same clade, presumably because these are cases of iciHHV, and parents always pass on the same variant of HHV to their offspring. HHV-6B also exhibits genetic diversity, with genomes in many different clusters that are less distinct than those of HHV-6A (Fig. 4B). Notably, samples with likely iciHHV-6B do always fall into the same clade as their family members, and the HHV genomes from these families are also very closely phylogenetically related to each other. HHV-7 also exhibits genetic diversity, and does not seem to originate from a single source (as might be the case if HHV-7 was a contaminant) (Additional file 1: Fig. S2). Interestingly, HHV-7 genomes from members of the same family tended to be much closer phylogenetically than HHV-7 from unrelated individuals (Mann–Whitney U test using distance matrix values, *p* value < .05). Removing the suspected iciHHV cases, HHV-6B also showed the same trend (*p* value < .05). This may indicate that the HHV-6B and HHV-7 variant that established itself in LCLs originated from an initial infection that was spread within a household.

### Several canonical telomeric sequences are integration sites for HHV 6A or 6B

**Fig. 5:**
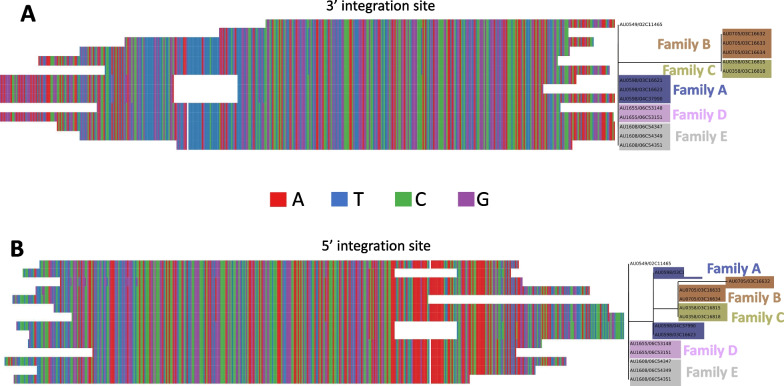
Phylogenetic trees and aligned sequences of assembled HHV-6A integration sites. **A** Assembled sequence of the HHV-6A 3’ integration site. **B** Assembled sequence of the HHV-6A 5’ integration site. Leaves are labelled with family and sample IDs <FAMILY ID>/<SAMPLE ID>. Suspected cases of iciHHV are highlighted, with coloring according to <FAMILY ID>

**Fig. 6:**
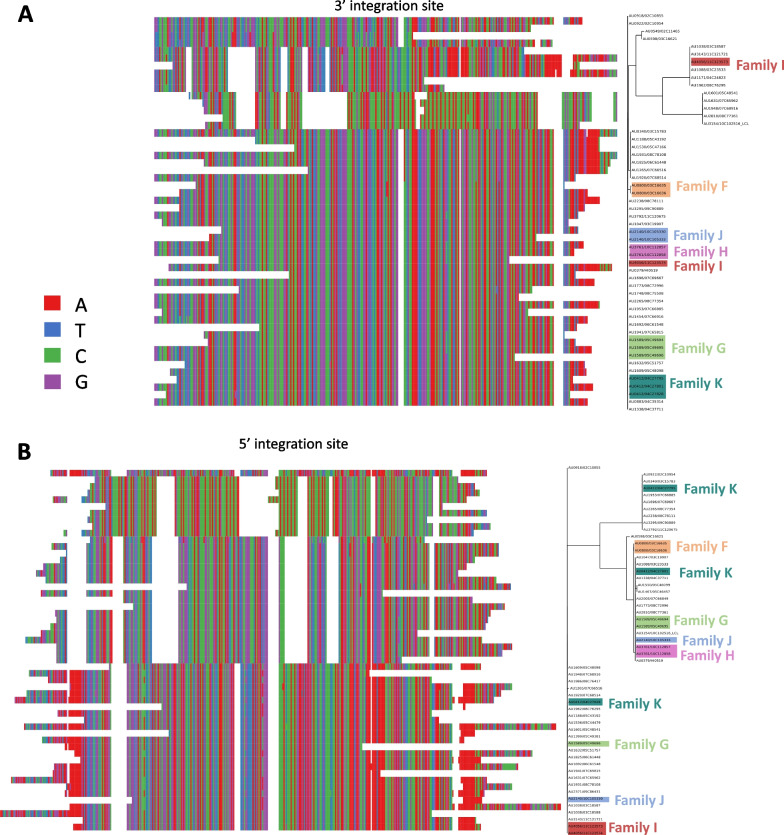
Phylogenetic trees and aligned sequences of assembled HHV-6B integration sites. **A** Assembled sequence of the HHV-6B 3’ integration site. **B** Assembled sequence of the HHV-6B 5’ integration site. Leaves are labelled with family and sample IDs <FAMILY ID>/<SAMPLE ID>. Suspected cases of iciHHV are highlighted, with coloring according to <FAMILY ID>

Similarly, we wished to analyze the genetic diversity of the different integration sites for HHV 6A and 6B. Using megahit, MAAFT, and ClustalW, we de novo assembled, aligned, and built a phylogenetic tree from the reads that did not align to HHV-6A, 6B, or 7 but had mates that aligned to HHV. HHV-7 had very few of such reads and thus de novo assembly was not possible in any sample. However, HHV-6A and HHV-6B show clear canonical flanking sequences, which we refer to as candidate integration sites (Figs. 5, 6). Interestingly, there is little variation within the 5’ and 3’ integration sites for HHV-6A (Fig. 5). Small single-nucleotide differences are shared among family members, indicating inherited integrated viruses and sites.

HHV-6B 5’ and 3’ flanking regions also cluster into clear canonical candidate integration sites. Both the 3’ and 5’ sites cluster into 3 distinct clusters, with highly dissimilar sequences (Fig. 6). Family members with suspected iciHHV-6B usually fall within the same cluster, however in the 5’ flanking integration site families AU0412, AU2140, and AU4056 fall into separate clusters and in the 3’ flanking region members from family AU4056 falls into separate clusters.

When we matched the candidate integration sites to public sequences using NCBI’s BLAST, all sequences matched to isolate HHV or endogenous HHV sequences. In particular, sequences matched to studies studying integrated HHV diversity [45, 47–50].

## Discussion

Using whole genome sequences, we extensively catalogue DNA viruses present in human whole blood and lymphocytes. Additionally, we found several viruses that are often transmitted within families. In particular, erythroviruses and torque teno viruses may be transmitted within households though the mechanism of the particular transmissions in our dataset remains unknown. Previous studies have identified both transplacental and fecal-oral modes of transmission in torque teno viruses [51]. Erythroviruses also can be transmitted transplacentally, and more commonly through respiratory droplets [52]. We additionally identified 28 cases of suspected iciHHV-6. We show that integrated herpesviruses are genetically diverse, with variable genomes sites and integration sites across families. Additionally, herpesviruses seem to be often transmitted within families, as samples from family members more often contain the same exogenous HHV-6B and HHV-7 variant than those from unrelated individuals. It may also be that common variants within families are the result of variation in herpesviruses specific to different regions in the U.S. [49]. HHV has been implicated in several diseases such as multiple sclerosis, encephalomyelitis, and febrile convulsions [53]. Genetic differences in exogenous HHV and iciHHV and its integration sites could influence disease pathology and contribute to different incidences of disease across different regions of the world [54] The unmapped read space of whole genome sequencing data is an easy method for better understanding HHV diversity and its possible role in disease.

To our knowledge, this is the first study to show evidence of widespread latency and possible integration of non-inherited HHV-6B and HHV-7 in LCLs. Moreover, previous studies have shown that HHV-6 and HHV-7 typically preferentially infect (CD4+) T-lymphocytes. However, the LCLs from the iHART dataset are derived from B-lymphocytes, indicating that B-lymphocytes may be an understudied route for HHV infection.

The patterns in our dataset suggest de novo integration of HHV-6B and de novo integration and then excision and reactivation of HHV-7 in LCLs. We hypothesize a primary infection of HHV-6B or HHV-7 in one or more lymphocytes from the donor de novo integrated into the host chromosomes, either while still in the host or during the process of LCL immortalization and storage. Genetic drift, positive selection, or reactivation then increased the fraction of cells with an integrated virus over time, leading to varying loads of HHV-6B and HHV-7 across samples. Alternatively, it is possible that HHV-7 established latency via an extrachromsomal nuclear episome that co-localizes to the chromosomes in order to replicate in tandem with the host cell. This is the life cycle of the related herpesvirus Karposi’s sarcoma herpesvirus (HHV-8) [55], though the loss of the DRs int he HHV-7 genome suggest that an integration and excision event did occur at some point in the HHV-7 life cycle.

In this study, we have used the unmapped read space of whole genome sequences to better understand prevalence and intra-family transmission patterns of various blood viruses. To our knowledge this is the first study using large WGS datasets of families in order to study viral transmission. Additionally, the unique family structure of our dataset allowed us to identify likely cases of iciHHV-6A and iciHHV-6B and document the genetic and integration site variation within these species. This is also the first study to observe and hypothesize about the widespread de novo HHV-6B and HHV-7 integration in LCLs. We hope this encourages further research on HHV-6 and HHV-7 integration and latency. The biological samples in our dataset with these unique distributions are available for future research upon request and application.

We performed such analyses using a collection of WGS data that was generated for unrelated purposes (to understand the genetic components of autism). We suspect whole genome sequences contain a wealth of untapped data, and may be valuable resources beyond their traditional GWAS use cases. Particularly, as more WGS data is generated from diverse global populations, the unmapped read space could be used to track the spread and geography of various viruses.

## Methods

### Dataset and original alignment to GRCh38

We obtained Whole Genome Sequencing (WGS) data from the Hartwell Autism Research and Technology Initiative (iHART) database, which includes 4842 individuals from 1050 multiplex families in the Autism Genetic Resource Exchange (AGRE) program [30]. A total of 4568 individuals from 1004 families passed quality control and were included in the analyses. DNA samples were derived from whole blood (WB) or lymphoblastoid cell lines (LCL) and sequenced at the New York Genome Center.

All WGS data from the iHART database have been previously processed using a standard bioinformatics pipeline which follows GATK’s best practices workflows. Raw reads were aligned to the human reference genome build 38 (GRCh38_full_analysis_set_plus_decoy_hla.fa) using Burrows-Wheeler Aligner (bwa-mem).

### Extracting unmapped and poorly unmapped reads

We excluded secondary alignments, supplementary alignments, and PCR duplicates from downstream analyses. We extracted reads from the iHART genomes that were unmapped to GRCh38 and the decoy reference or mapped with low confidence. Low-confidence reads were defined as reads marked as improperly paired and had an alignment score below 100. We used alignment score rather than mapping quality in order to select for reads were likely not true alignments to the human reference genome, rather than for reads that had ambiguous alignments to GRCh38. These reads were then re-paired if both ends needed to be realigned, and lastly separated into single-end and pair-end files.

### Taxonomic classification and aggregation

We used Kraken2 [56] to align the unmapped and poorly aligned reads to a the Kraken default (RefSeq) databases of archaeal, bacterial, human (GRCh38.p13), and viral sequences [57]. These reference databases were accessed on Feb 16, 2021. Kraken2 was run on the unmapped and poorly mapped reads from each sample, using the default parameters. Because Kraken was able to map the majority of reads down to the species or strain level, Kraken classifications were aggregated by species before downstream analysis.

### F-regression on metadata

To analyze the effect of various demographic (such as household, autism status, and sex) and experimental parameters (such as sequencing plate and sample type) on microbial and viral profile, we performed an F-regression analysis. We chose an F-regression because many variables were highly collinear with each other: for example, samples from the same household were nearly always sequenced on the same sequencing plate, autism is much more prevalent in males, and the same sample types were normally collected from households. For each microbe, we built an ordinary least squares (OLS) model, using as our regressor an indicator matrix of sample type, sex, child vs. parent, autism status, sequencing plate, household/family, and sample id, and as our response variable the log-normalized counts of microbes (with pseudo-counts of 1). Using the statsmodels library, we then ran a forward OLS regression in which we iteratively selected the regressor features that best explained the previous models residuals, and ceased adding features when the ANOVA score between the previous and new models was no longer statistically significant (adjusted *p* value<.05).

### Realignment to herpesvirus reference genomes

Using bwa-mem with the default parameters, we aligned all reads classified by Kraken as belonging to herpesviruses to a set of reference genomes consisting of GRCh38 and the decoy, and all the herpesvirus genomes present in the RefSeq database. Most importantly, this included human betaherpesvirus 6A (NC_001664.4), human betaherpesvirus 6B (NC_000898.1), human betaherpesvirus 7 (NC_001716.2 ), and both the decoy and RefSeq genome for human gammaherpesvirus 4, or the Epstein–Barr virus (chrEBV in the decoy genome, and NC_007605.1 in RefSeq). We performed the same analysis using 2504 high-coverage WGS LCL samples from the most recent release of the 1000 genomes dataset (Additional file 1: Fig. S3).

### HHV read counts, paired-end analysis, and coverages

To covert the herpesvirus read counts to viral genomes per host genome, we normalized against the average coverage for two housekeeping genes are not known to show copy number variation, EDAR and HBB [58].

We used pysam and an in-house script to collect genome-wide coverages for different combinations of pairings in order to generate the coverage graphs in Figs. 2 and 3.

### De novo assembly and clustering of HHV viral genomes and integration sites

To generate the integration site assemblies and alignments (Figs. 5, 6), we first extracted reads that were not classified as herpesvirus reads but had a mate that aligned to the start or end of the herpesvirus genome. For each individual, we de novo assembled these reads. Using MAAFT [59], we then performed multiple sequence alignment of these assemblies, and used ClustalW to generate phylogenetic trees. We used the default parameters for MAAFT, and allowed for reverse complementary sequences to be generated as needed. Before generating phylogenetic trees, we attempted to remove redundant sequences that might correspond to a forward sequence and its reverse complementary sequence. We performed the following logic: if a sample had two assembled sequences (presumably corresponding to a forward sequences and a reverse complementary sequence), we removed the sequence that had the least number of matches to the consensus sequence generated by all samples. We used ClustalW [60] on the EMBL browser [61], with a neighbor-joining algorithm, no distance correction, and ignoring gaps.

We BLASTED these assembled sequences against NCBI’s nt nucleotide collection using the default parameters, and not masking low-complexity regions.

To generate the assemblies of the viral genomes, we extracted reads aligned to HHV-6A, HHV-6B, and HHV-7. We used bcftools to perform variant calling on all of the samples against the reference HHV-6A, HHV-6B, and HHV-7 genomes. We used VCF2phylip to convert the variant calls to alternate reference sequences. We filtered to samples that had variants or reference alleles called for at least 50% of loci. Similar to the integration sites, we performed multiple sequence alignment on the reconstructed viral genomes using MAAFT with the default parameters and generated phylogenetic trees using ClustalW using the same parameters as above. [62]

We used Biopython’s Phylo library [63] an in-house python script to generate the sequence alignment trees and diagrams used in Figs. 4, 5, 6 and Additional file 1: S2.

## Supplementary Information

**Additional file 1.** Supplementary figures depicting the abundances and associations of common viruses (S1), the phylogenetic tree for HHV-7 (S2), correlations between counts of different herpesviruses (S3), and the number of reads aligned to herpesviruses in the 1000 genomes dataset (S4).

## Data Availability

Analysis code and scripts, as well as the sequences used for KRAKEN classification and herpesvirus realignment can be found at https://github.com/briannachrisman/blood_microbiome. The raw reads from the iHART samples can be found on Anvil, maintained by NHGRI at https://anvilproject.org/data/studies/phs001766. Dataset access is controlled in adherence to NIH Policy and in line with the standards set forth in the individual consents involved in each cohort.
