# Supplementary material for: Transmission dynamics of human herpesvirus 6A, 6B and 7 from whole genome sequences of families
Source: Virol J. 2022 Dec 24;19:225. doi: 10.1186/s12985-022-01941-9 (PMC9789512; doi:10.1186/s12985-022-01941-9)
Supplement: Supplementary file 1 — Additional file 1. Supplementary figures depicting the abundances and associations of common viruses (S1), the phylogenetic tree for HHV-7 (S2), correlations between counts of different herpesviruses (S3), and the number of reads aligned to herpesviruses in the 1000 genomes dataset (S4). [file 12985_2022_1941_MOESM1_ESM.pdf]

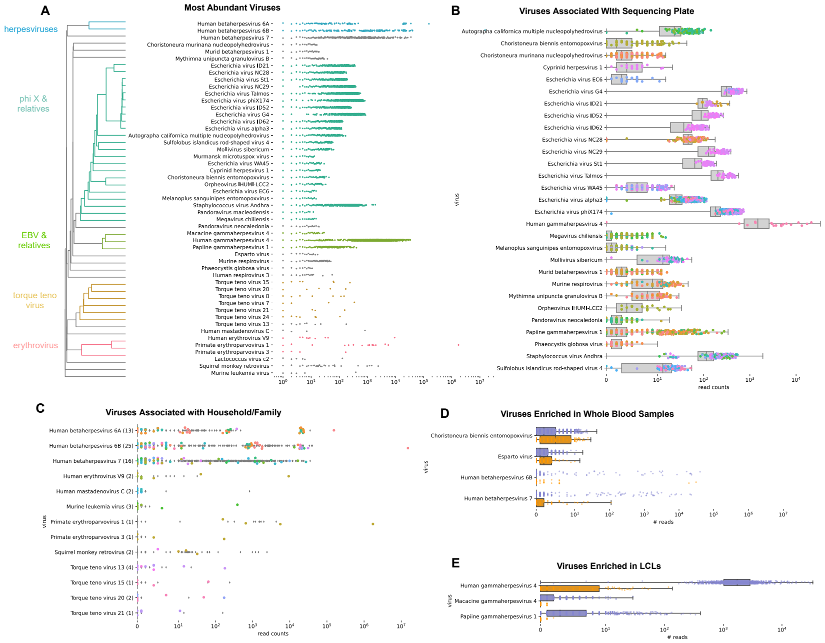


Figure S1: **(A)** Top 50 most abundant viruses, clustered by Spearman correlation across samples. **(B)**Viruses with abundance significantly associated with sequencing plate, per F-regression results. Colors represent sequencing plates that had significantly higher abundances of a given microbe compared to the rest of the population. Samples from sequencing plates without significant enrichment of a microbe are captured in the grey box plots. **(C)** Viruses associated with household per F-regression. Like **(B)**, colors represent households that had significantly higher abundances of a given microbe compared to the rest of the population. Samples from households without significant enrichment of a microbe are captured in the grey box plots. Note that a non-visible boxplot indicates that the median, 5% and 95% percentiles were at zero. **(D)** Viruses associated with cell type per F-regression results, and more abundant in whole blood samples (orange) than LCL samples (purple). **(E)** Viruses associated with cell type per F-regression results, and more abundant in LCL samples than whole blood samples.

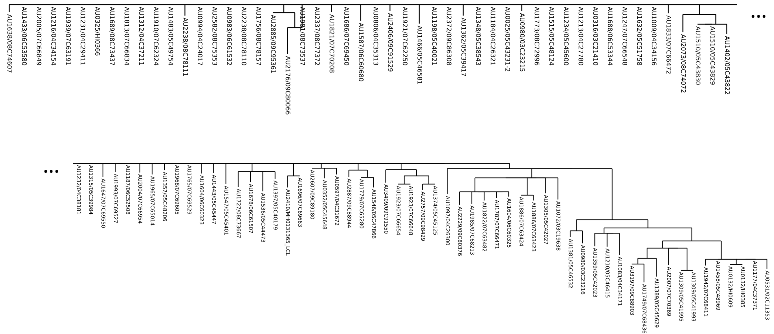

Figure S2: Phylogenetic tree of *de novo* assembled HHV 7 genomes from the samples where *de novo* assembly was successful. Leaves are labelled with family and sample IDs <FAMILY ID>/<SAMPLE ID>.

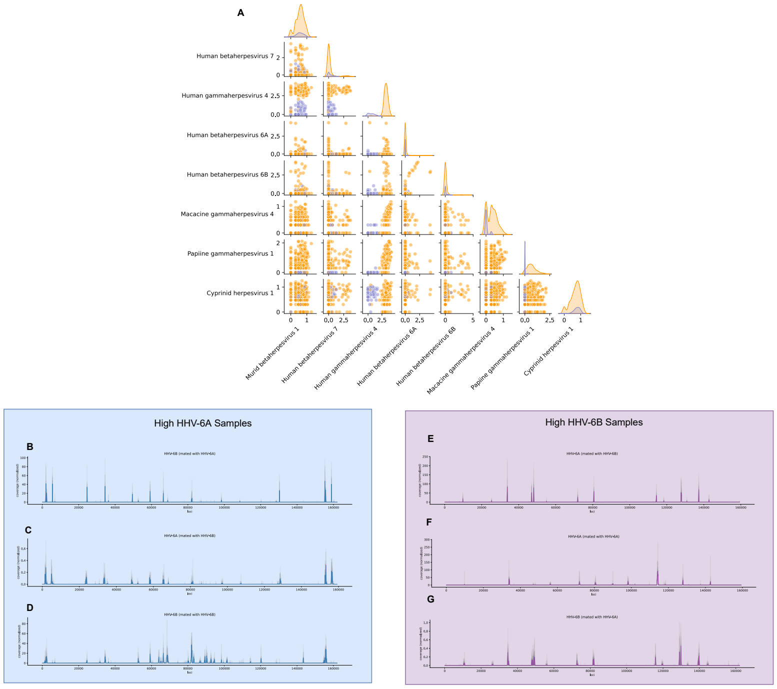


Figure S3: **(A)** Pair-plots of herpesviruses counts. There are some slight correlations due to mismappings between homologous and low-complexity regions. **(B)-(D)** Normalized coverages for samples with high HHV-6A loads. **(B)** Coverage of reads mapped to HHV-6A that were paired with mates mapped to HHV-6B. **(C)** Coverage of reads mapped to HHV-6B that were paired with mates mapped to HHV-6A. **(D)** Coverage of reads mapped to HHV-6A that were paired with mates mapped to HHV-6A. **(E)-(G)** Normalized coverages for samples with high HHV-6B loads. **(E)** Coverage of reads mapped to HHV-6B that were paired with mates mapped to HHV-6A. **(F)** Coverage of reads mapped to HHV-6A that were paired with mates mapped to HHV-6B. **(G)** Coverage of reads mapped to HHV-6A that were paired with mates mapped to HHV-6B.

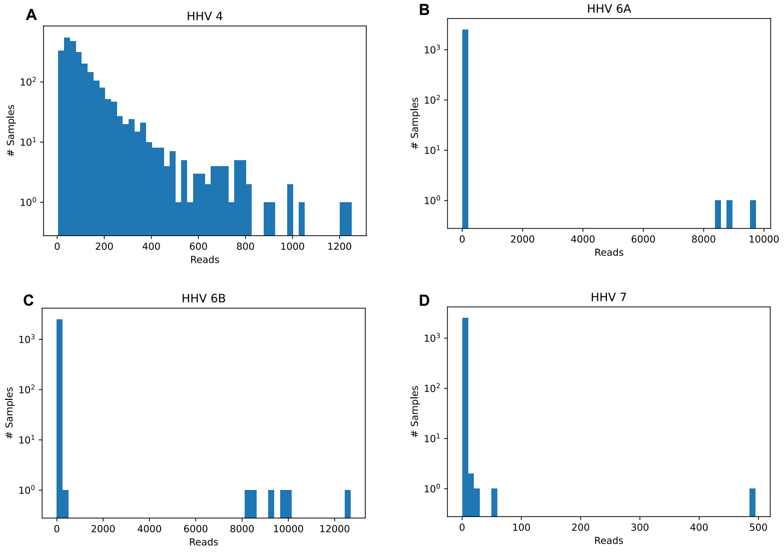


Figure S4: **(A)** Numbers of reads aligned to HHV-4 (Epstein Barr Virus) in the 1000genomes dataset. **(B)** Numbers of reads aligned to HHV-6A in the 1000genomes dataset. **(C)** Numbers of reads aligned to HHV-6B in the 1000genomes dataset. **(D)** Numbers of reads aligned to HHV-7 in the 1000genomes dataset.
